# Disruptive supply chain technology assessment for sustainability journey: A framework of probabilistic group decision making

**DOI:** 10.1016/j.heliyon.2024.e25630

**Published:** 2024-02-08

**Authors:** Humaira Nafisa Ahmed, Sayem Ahmed, Tazim Ahmed, Hasin Md Muhtasim Taqi, Syed Mithun Ali

**Affiliations:** a Department of Industrial and Production Engineering, Bangladesh University of Engineering and Technology, Dhaka, 1000, Bangladesh; b Department of Mechanical and Production Engineering, Ahsanullah University of Science and Technology, Dhaka, 1208, Bangladesh; c Department of Industrial and Production Engineering, Jashore University of Science and Technology, Jashore, Bangladesh

**Keywords:** Bayesian-BWM, Disruptive technologies, Emerging economy, Internet of things, Sustainable supply chain

## Abstract

The fourth industrial revolution, commonly recognized as Industry 4.0, has been ushered by modern and innovative intelligence and communication technologies. Concerns about disruptive technologies (DTs) are beginning to grow in developing countries, despite the fact that the trade-offs between implementation difficulties and realistic effects are still unknown. Hence, prioritization and promotion of such technologies should be considered when investing in them to ensure sustainability. The study aims to provide new critical insights into what DTs are and how to identify the significant DTs for sustainable supply chain (SSC). Understanding the DTs’ potential for achieving holistic sustainability through effective technology adoption and diffusion is critical. To achieve the goal, an integrated approach combining the Bayesian method and the Best Worst Method (BWM) is utilized in this study to evaluate DTs in emerging economies' supply chain (SC). The systematic literature review yielded a total of 10 DTs for SSC, which were then evaluated using the Bayesian-BWM to explore the most critical DTs for a well-known example of the readymade garment (RMG) industry of Bangladesh. The results show that the three most essential DTs for SSC are “Internet of things (IoT)”, “Cloud manufacturing”, and “Artificial intelligence (AI)”. The research insights will facilitate policymakers and practitioners in determining where to concentrate efforts during the technology adoption and diffusion stage in order to improve sustainable production through managing SC operations in an uncertain business environment.

## 1 Introduction

The prosperity of disruptive technologies (DTs) encourages the surge of adopting new ways of sustainable production, posing further challenges to organizations of emerging economies due to the highly competitive and continuing globalization of the market world [1]. The adoption and implication scope of DTs needs to be evaluated using a structural assessment framework on the basis of sustainability concepts i.e., economic, environmental, and social [2]. With the rapid growth of DTs, today's readymade garments (RMG) of emerging economies face the urgency to change their product creation procedures in their supply chain (SC) to improve economic growth and performance, and ensure resilience while still focusing on environmental and social values. The RMG industry is one of Bangladesh's most significant accelerators for economic and social growth, which currently subsidizes 11.2 percent GDP of Bangladesh [3]. However, RMG have been dealing with unprecedented disruption as a result of the COVID-19 pandemic. To overcome this interacting with all stakeholders is one of the salient aspects of creating a resilient SC that seeks to be adaptive and agile [4]. In a SC, sharing information is the most effective approach to enhance visibility and decrease risks [5]. To be more flexible and resilient to disturbances, all the stakeholders of SC in RMG should redesign uncertainty or risk management as part of their routine in such a way that the focus does not move away from sustainable operations in the event of disruptions. However, using DTs in a sustainable supply chain (SSC) causes a revolution in various industries, resulting in competitive advantages for businesses and an improvement in consumers' quality of life [6]. A SSC is described by Carter & Rogers (2008) as the alignment of key business processes with SC partners to achieve environmental, social, along with economic sustainability goals [7]. Because of its focus on the economic goals of organizations with less adverse effects on the climate, sustainability has recently become a hot topic among academics and practitioners. Along with sustainability, organizations of emerging economies must adhere to the 17 Sustainable Development Goals (SDGs) founded by the United Nations (UN) in 2015 for the year 2030 [8]. To maximize the benefits of DT adoption and diffusion for overall sustainability, each DT must be carefully analyzed since different DTs can have varying effects on enterprise and sustainability aspects depending on the SDGs [2].

Over the recent years, the fourth industrial revolution has given rise to breakthrough DTs in all business models, including the sustainable supply chain management (SSCM) field [9]. Technology disruption, according to Sood & Tellis, (2011), occurs when a new technology outperforms the leading technology on the key dimension of efficiency, implying that disruption may occur on various scales and has the potential to significantly increase productivity [10]. Industry 4.0 introduced different DTs, which are able to improve the SSCM operations models and enhance the efficiency of organizations in uncertain business environments. These technologies have been described as the most successful means of enhancing sustainability and increasing company competitiveness [11]. To name a few of these technologies, blockchain, internet of things (IoT), big data analytics (BDA), etc., have substantial impacts on today's competitive SC to enhance its visibility and traceability. For instance, blockchain based services provide a more safe, robust, and reliable solution with improved traceability compared to existing technology or management systems [12]. Blockchain technology can provide a secure and transparent platform for sharing information, verifying certifications, and ensuring ethical and sustainable practices. IoT technologies, using modern wireless telecommunications incorporate interaction and collaboration between individuals and devices along with the internet, which can provide the SC opportunities to track every phase of the goods or services, from the design of commodities to the after-sales operation, in real-time [13]. IoT sensors can monitor energy usage in factories, identify areas for improvement, and enable real-time energy management. BDA can be used to examine the massive amount of information produced by IoT to find flaws and vulnerabilities in the SC [14]. Technologies like RFID tags on garments can provide consumers with detailed information about the product's origin, materials, and manufacturing processes. While these information and communication technologies are mainly digital, many physical DTs, such as additive manufacturing, collaborative robots, and drone delivery system, have also complimented manufacturing operation systems with higher quality, improved energy consumption, and resource efficiency in SSC.

As SCs have become increasingly complicated, consumers require more product safety, quality, and sustainability information. As the demand for new types of products escalates, companies are called to supply sustainable products and services to satisfy ethical customers. Due to customers' changing preferences and lifestyles, it is critical for organizations to alter their business processes to remain up-to-date to utilize these technologies [15]. To put it another way, businesses must become increasingly flexible and responsive to modify the SC in light of changing customer demands. Despite the greater long-term technological consequences on society and society's sustainability goals, technologies still require more attention as well as evaluation [16]. These technologies may be difficult to analyze using typical assessment techniques; however, extra review for sustainability advantages may help them acquire strategic approval. Overall, effective rigorous assessment methods and relevant tools of evaluation can assist in implementing and understanding those technologies and the longer-term effects they have on organizations.

One of the key goals of this research is to investigate links between SCs, DTs, and sustainability while dealing with business uncertainty. The second is to strive for value creation in the SC through disruptive technological solutions in the economic, social, and environmental perspectives of sustainability. The research context of DT in SSC focuses on examining how DT adoption and integration might lead to socially and environmentally responsible SC practices. DTs provide opportunities to enhance supply chain sustainability (SCS) by improving transparency, optimizing operations, reducing waste, and minimizing environmental impact. By leveraging these technologies, businesses can make their SC more efficient, resilient, and environmentally friendly. According to the authors' understanding, there are few studies that combine SC, DTs, and sustainability. Furthermore, while numerous types of research have been directed at the implementation of specific DTs in various areas, there is a dearth of research on the approach to evaluating value through DTs in every dimension of sustainability. The key focus of this research is to identify the crucial DTs to ensure SCS in emerging economies and explore the answer to the following research questions.

RQ 1 What are the DTs that can facilitate sustainability in SC, and how can they contribute to achieving SDGs?

RQ 2 What are the significant and crucial DTs for improving sustainability of emerging economies' SC?

RQ 3 How do SC leaders and policymakers comprehensively evaluate the DTs for successful implementation in uncertain business environment?

Multi-echelon, geographically disjointed companies compete to satisfy customers in today's SCs, which are increasingly complex. In addition, there are new stresses on SC practice and policy to contemplate and endorse SCS. Such concerns raise the question of whether conventional technologies are capable of supporting the SSC needed for the appropriate provenance of products and services in a safe, transparent, and reliable manner. New technologies and approaches make these objectives more pragmatic and economically viable. The study aims to examine and assess the effect of DTs on the creation of sustainability. Therefore, this study assesses the critical DTs for enhancing SCS using an integrated approach combining Bayesian Method and Best Worst Method (BWM). The DTs weights are measured through the Bayesian best-worst equation. Given that the decision-makers inputs are collective, a probabilistic approach to determining the aggregated weights of DTs is optimal. Since Bayesian-BWM is constructed following the conventional BWM, the input, namely pairwise judgments, is identical. Nevertheless, there is a distinction between the two approaches in terms of performance. The Bayesian-BWM method provides us with a more sophisticated and statistically rigorous approach by allowing for the integration of prior information and quantification of uncertainty. The final production of the original BWM is a concrete weight value, while the Bayesian-BWM offers a probability distribution along with a hierarchical probabilistic relationship among the technologies. The Bayesian-BWM enhances the traditional BWM by providing a more robust, flexible, and statistically rigorous approach to capturing and analyzing preferences. The application of BWM is prominent in various areas. However, less research has been done on an integrated Bayesian-BWM process, specifically on assessing essential DTs to every dimension of sustainability in the SC of an emerging economy. The contribution of this research can be outlined as follows.

i. Investigating the relationships amid DTs, SC, and sustainability in an emerging economy context.
ii. Exploring a comprehensive list of DTs from a thorough literature review in an emerging economy concept.
iii. Formulating a Bayesian-BWM to rank the crucial DTs of SSC.
iv. Aiding policymakers in making rational choices when assessing essential DTs in an emerging economy.

This study is divided into six parts, the first of which contains an introduction to SSC and DTs. Section 2 explains the research background of this study. A comprehensive framework of Bayesian-BWM is described in Section 3. The results followed by findings are analyzed and discussed with implications in section 4 and section 5, respectively. Finally, the final chapter discusses the limitations as well as future scopes.

## 2 Literature review

New technologies have the potential to revolutionize the business's operation, and SC must be restructured on a regular basis to keep up with evolving dynamics [17]. While restructuring the SC, sustainability must be considered. It's no secret that researchers and experts alike are involved in learning more about the benefits of implementing SSCM [18,19]. SSCM may be characterized as an approach to managing the flow of information, resources (materials and money), collaboration between SC partners, and the triple-bottom-line (TBL) components [20]. Several important global forums have debated sustainability regularly to ensure corporations recognize the necessity of achieving sustainability in their business operations [21]. For instance, Sánchez-Flore et al. (2020) directed a methodical research of how sustainability affects SC efficiency from the perspective of an emerging economy [22]. Hence, the integration of the three sustainability pillars and their impact on SC performance is crucial from an emerging economy viewpoint. Queiroz et al. (2021) conducted a study regarding DT namely blockchain adoption behaviors and barriers in SC for emerging economy [23]. Findings from their research suggest that the most important constructs are enabling circumstances, trust, social influence, and effort anticipation. Meanwhile, Kumar et al. (2020) examined the behavioral factors for adopting SSC practices [24]. The results indicate the most influential behavioral elements, 'organization culture', and 'higher-level commitment'. In addition, Khan et al. (2021) evaluated the current and growing trends in the area of SSCM, as well as future research directions, while conducting a literature review [25]. Research methodologies based on multiple-criteria decision-making (MCDM) and firm-level studies have predominated in this domain, according to their findings.

In recent years, studies on DTs are getting increased consideration by researchers and experts [26,27]. DT shakes up a SC and has the potential to completely change how products are manufactured, distributed, and tracked [28]. For instance, Paliwal et al. (2020) explored the importance of the DTs in the SSC and found the major benefits of adopting DTs are traceability and transparency [29]. Moreover, Lekan et al. (2020) directed a study to investigate the intervention of DTs to accomplish SDGs and recommended the route for the deployment and achievement of the SDGs through disruptive innovations [30]. Furthermore, DTs are promising and essential for sustainable future education [31]. Research has shown that educational technology can substantially increase education and learning when applied appropriately. Dolgui & Ivanov (2020) addressed recent advances in SC structural dynamics research, focusing on positive (i.e., modern DTs) structural dynamics triggers in complex supply chain network (SCN) [32]. Abdel-Basset et al. (2021) presented a framework using DTs for COVID-19 investigation [33]. They also advised policymakers and the government on how to implement DTs to mitigate the effects of COVID-19 outbreaks. In order to increase vulnerable resilience capacity under the COVID-19 pandemic, Ali et al. (2021) defined 14 capability elements and their sub-factors in the RMG business in Bangladesh [34]. They stated DT such as BDA plays a vital role in SC resilience. So, it is very important to assess the DTs and find the relationship between DTs and SSC. Therefore, Table 1 shows the list of possible DTs of SSC and the relationship of DTs with sustainable dimension.

**Table 1:** List of disruptive supply chain technologies.

Code	Technology Name	Relationship with sustainable supply chain	Relationship with Sustainable Dimension			References
			Social	Economic	Environment	
T1	Big data analytics (BDA)	The application of BDA helps organizations to formulate SC strategies and increase the degree of customization, as well as the level of customer service that eventually improves the customers' satisfaction and social sustainability.	✓	✓		[35]
T2	Internet of things (IoT)	IoT allows businesses to play a crucial role in proceeding with sustainable growth by reducing carbon emissions and use of non-renewable resources. Moreover, IoT ensures a win-win situation by creating a viable business model for economic development through connecting people and organizations.	✓	✓	✓	[[36], [37], [38]]
T3	Blockchain technology	The economic aspects of blockchains offer new opportunities, higher operating efficiency, and greater savings. The social empowerment of SC can be realized through blockchain's ability to build trustworthy relationships among SC participants. Blockchain also has the ability to expand the efforts in the environmental policy and strategy by cutting down the strain on energy and natural resources.	✓	✓	✓	[39,40]
T4	Additive manufacturing	Additive manufacturing i.e., 3D Printing could turn into a multi-dimensional tool that offsets many negative environmental impacts of the molding and manufacturing processes that use a lot of energy, while minimizing the risk of manufacturing waste. It is unleashing a booming manufacturing sector and boosting productivity, which, in turn, would result in greater income for individuals and job growth.	✓	✓	✓	[41]
T5	Automation and collaborative robotics	Automation and collaborative robotics ensure less human engagement, which reduces production time and improves productivity.		✓		[42]
T6	Artificial intelligence (AI)	AI has the advantage of enabling decision support systems to be developed to carry out supplier selection, introduce agility, and explore big data trends in order to identify risks and manage information in the uncertain business environment.		✓		[40]
T7	Drone	Drones are critical for delivering information and products, which could greatly accelerate the delivery system. Drones are environment friendly because they are operated by solar system. In addition, organizational activities can easily be monitored with drone systems which may ensure proper security.		✓	✓	[43]
T8	Radio frequency identification (RFID)	RFID based SCs mitigate disruption which provides an organization with economic benefits. It is considered a promising technology for SC functions such as forecasting, inventory management, and retail operations, while simultaneously increasing performance, precision, visibility, and security.	✓	✓		[44]
T9	SC digital twin	A digital twin SC provides better efficiency, greater productivity, less expense, and greater versatility and sustainability in manufacturing.		✓	✓	[45,46]
T10	Cloud manufacturing	Cloud manufacturing is a services-oriented approach to sharing manufacturing tools and skills on a cloud-based network. Cloud manufacturing offers flexibility and enhances efficiency through the optimum allocation of resources and reduction of waste. It also decreases the go-to-market periods.		✓	✓	[47]

The above studies show that SSC would increase its efficiency if the organization assesses DTs and introduces DTs in practice. Therefore, it is important to assess DTs. The extant literature showed several MCDM approaches i.e., Best Worst Method (BWM), Decision Making Trial and Evaluation Laboratory (DEMATEL), Technique for Order Preference by Similarity to Ideal Solution (TOPSIS), etc., have been followed to evaluate possible alternatives i.e., investigating the social sustainability of SC [48], assessing service performance of airline industry [49], assessing resilient SC [50] and evaluating the significance of logistics performance measures [51].

Moktadir et al. (2020) used the BWM to evaluate sustainable threats to the emerging economy's leather SC [52]. Their study showed that the absence of a financial facility is a key obstacle to implementing circular economy practice successfully. Kouhizadeh et al. (2021) ranked the blockchain adoption barriers for SSC by using the DEMATEL method [53]. To select and rank the sustainable waste disposal technologies for municipal waste management, Torkayesh et al. (2021) developed an integrated stratified MCDM and BWM [54]. Later, Moreno-Solaz et al. (2023) applied a Stratified-BWM in selecting waste collection trucks considering sustainability and uncertainty associated with feasible future scenarios [55]. To evaluate sustainable and resilient IoT supplier selection, Bonab et al. (2023) proposed an integrated approach based on spherical fuzzy and BWM to reduce uncertainty in pairwise comparisons [56]. Dong et al. (2021) implemented a fuzzy BWM based on Bellman and Zadeh's extension principle for MCDM problem and compared their findings with BWM and conventional fuzzy BWM [43]. Several authors proposed the Bayesian-BWM for introducing the group decision-making problem. For instance, Mohammadi & Rezaei (2020) proposed a Bayesian-BWM approach for the probabilistic decision-making model [57]. Their suggested technique reads the BWM's input using probability distributions, which keeps the BWM's basic premise unchanged. In addition, Li et al. (2020) presented a Bayesian-BWM to analyze crowdsourcing delivery personnel and they found “Skills” as the most important criteria [58]. Later, Aghajani Mir et al. (2022) proposed a Bayesian-BWM approach for identifying and prioritizing challenges of implementing blockchain technology in the supply chain [59]. Furthermore, Ak et al. (2022) assessed occupational risk in textile production using the hybrid Bayesian-BWM method. They found electricity hazard as the highest risk rating from their study [60].

Two approaches for assigning weights on criteria or attributes in the context of MCDM are subjective weighting and objective weighting. The choice between subjective and objective weighting depends on the specific context of the decision problem, the availability of data, and the preferences of the decision-makers. Subjective weighting is used for criteria that are difficult to quantify or where expert judgment is valuable, and objective weighting is a more data-driven approach that relies on quantitative information and empirical evidence to determine criteria weights. Objective weighting is mostly represented by principal component analysis [[61]] and the entropy weight approach [][62] and is based on the data properties of each evaluation object. Subjective weighing techniques include-analytic hierarchy process (AHP), analytical network process (ANP), DEMATEL, TOPSIS, BWM, Stratified BWM, Bayesian BWM. Table 2 shows a quick comparison of the various MCDM methods.

**Table 2:** Comparison of the various MCDM methods.

Attribute	AHP	ANP	DEMATEL	TOPSIS	BWM	Stratified BWM	Bayesian BWM
Approach	Hierarchical	Network	Causal	Ranking	Scoring	Scoring	Probabilistic
Key Features	Hierarchy of criteria and sub-criteria	Accounts for interdependencies	Identifies cause and effect relationships	Ranks alternatives based on closeness	Assess alternatives based on best-worst	Stratification of the attributes considering feasible future scenarios	Incorporates Bayesian probability and the conditional independence of different attributes
Uncertainty	Not considered	Not considered	Not considered	Not considered	Not considered	Considered	Considered
Validation	Consistency check	Consistency check	Causal relationship analysis	None	None	Consistency check	Consistency check
Interdependency	No	Yes	Yes	No	Yes	Yes	Yes
Applicability	Various decision-making scenarios	Complex, networked decision problems	Understanding causal relationships and complex interrelations	Simple ranking and selection of alternatives	Intuitive and straightforward assessments	Decision making considering feasible future scenarios	Handling uncertainty in decision-making

[[54], [57], [63], [64], [65], [66], [67], [68], [69], [70]].

It is apparent from the debate above that DTs remain a new concept for emerging economies [22,23,52,71,72]. The acquisition and exploitation of different DTs in emerging economies are quite different than that of developed countries due to a lack of infrastructure, knowledge barriers, high investment costs, and reliance on labor. Hence, for the adoption of suitable DTs for an organization in emerging economies, the evaluation of DTs is necessary. Accordingly, it is important to differentiate between the critical and remarkable DTs for an emerging economy's SSC [73]. As of yet, no meaningful evaluation of DTs in emerging economies like Bangladesh has been conducted. In most studies, only a particular DT was simply addressed i.e., blockchain technology for business [39], review on artificial intelligence [74], etc. However, the interactions among the DTs and their applicability to SSC are yet to be explored. So, it is necessary to assess multiple DTs to choose the best and most suitable DTs for a corporation. Hence, assessing the DTs for an emerging economy remains a research gap. A Bayesian-BWM approach to the DTs of an emerging economy such as Bangladesh is suggested in order to address this void. For describing the partialities of a group of decision-makers, the proposed Bayesian-BWM is especially efficient. The vital contribution of this research is to examine the relationships amid DTs, SC, sustainability, and formulate a Bayesian-BWM approach to rank the crucial DTs.

## 3 Methodology

**Fig. 1:**
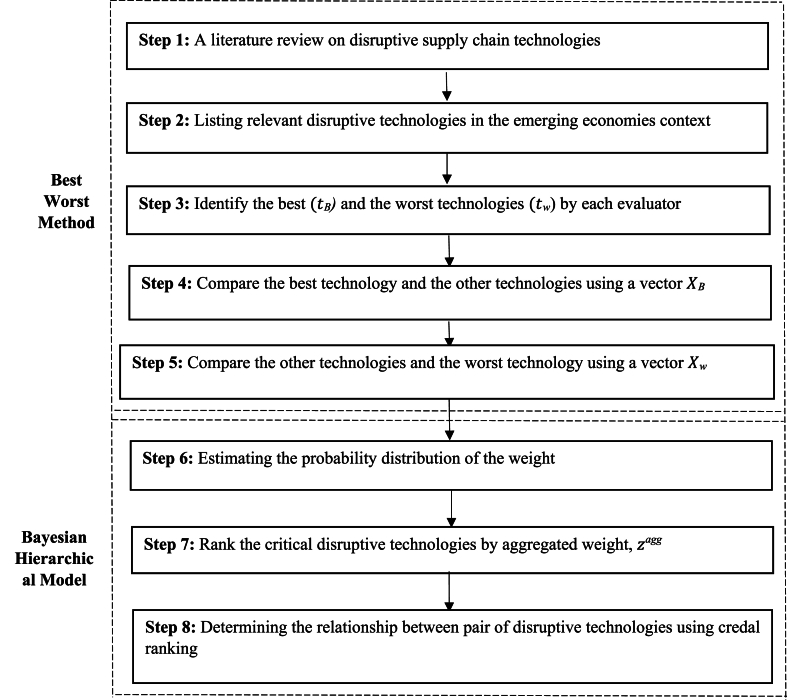
Detailed flowchart of the suggested methodology.

In this research, an integrated Bayesian-BWM is incorporated to prioritize DTs in organizations of emerging economies in order to promote SCS. This method takes into account all potential DTs that can improve sustainability in conventional SC and uses a probabilistic approach to assess the weight of the technologies, allowing them to be ranked. Prior to this, the Bayesian approach and BWM integration methodology proposed by Mohammadi & Razaei [57] have only been used in a few studies. A detailed framework of the suggested methodology is illustrated in Fig. 1.

### 3.1 Context and data collection

The proposed methodology (Fig. 1) has been implemented to assess DTs to achieve sustainable SC for the RMG industry of Bangladesh as an emerging economy. Many RMG companies in Bangladesh have sought to introduce and adopt various DTs to achieve SCS to tackle the disruptions resulting from the recent COVID-19 pandemic. However, most of them have failed to do so because of not having the proper understanding of the relationship between these DTs and the SCS issues while dealing with business uncertainty. Therefore, this study has considered this case to apply the proposed framework.

After reviewing the previous literature, 10 SC technologies have been listed as the disruptive SC technologies which will influence the SCS of the RMG industry of Bangladesh (Table 1). Assessment of these disruptive SC technologies requires high level of expertise and specialization in the field of SC systems and modern technologies as well as familiarity with the process of BWM. Therefore, this study set the expert inclusion criteria as strong domain knowledge, years of working experience, and familiarity with the BWM. Considering these inclusion criteria, the current study has selected 10 experts using the purposive sampling technique [75] as the evaluators of the disruptive SC technologies who have more strong expertise in SC technologies and the RMG industry, have more than 10 years of professional or academic involvement in the SC domain and have familiarity with group decision making using BWM. The profile of the evaluators is represented in Table 3. Given the specific inclusion criteria required for this study, small sample size was deemed necessary to ensure the selection of experts who met the stringent qualifications and expertise necessary for assessing disruptive SC technology. However, one advantage of using the Bayesian Best Worst Method (BWM) is its ability to overcome the limitation of small sample sizes. The BWM is designed to handle situations where the number of experts or respondents is limited, yet it still provides robust and meaningful results [,][[76], [77]]. Those who have been selected as experts have given their consent verbally to contribute to this research, and they responded to the questions seeking anonymity.

**Table 3:** Profile of the evaluators.

Evaluator	Designation	Experience	Expertise
E1	General Manager, Supply Chain	22 years	Supply chain management and RMG operations
E2	Associate Professor, Industrial and Production Engineering.	14 years	Supply chain sustainability
E3	Professor, Industrial Engineering and Management	17 years	Supply chain and operations management
E4	Senior Manager, Supply Chain	14 years	Sourcing and procurement
E5	Associate Professor, Industrial and Production Engineering.	12 years	Supply chain sustainability
E6	Senior Manager, Supply chain	13 years	Supply chain management
E7	Deputy General Manager, Supply Chain	11 years	Logistics and operations management
E8	Associate Professor, Industrial and Production Engineering	13 years	Production and supply chain management
E9	Senior Logistics Manager	16 years	Logistics and operations management
E10	Deputy Manager, Supply Chain	11 years	Supply chain management

A questionnaire was prepared for the evaluators for eliciting inputs and information to assess the importance of these DTs to achieve SSC for RMG industry. Then, the questionnaire was sent to each evaluator through email. They were communicated about the objective of the study. First, they were asked to determine the best technology and the worst technology among the 10 DTs based on their knowledge and experiences (see Table A1 in Appendix A of supplementary file). Then, they were questioned to determine the precedence score of the best technology over the other technologies (see Table A2 in Appendix A of supplementary file) as well as the precedence of all the technologies against the selected least important technology (see Table A3 in Appendix A of supplementary file) using the 1 to 9 scoring scale. In the next step, Bayesian-BWM was applied to finalize the ranking of the DTs in achieving SCS for RMG industry.

### 3.2 Best Worst Method

Among the numerous MCDM approaches available, BWM developed by Rezaei, (2015) was chosen to solve this problem due to some attractive inherent characteristics [65]. Since it does not require a complete pairwise judgment matrix, BWM needs less data and provides more reliable results due to its optimized pairwise comparison framework. At first, choosing the best as well as the worst technologies and then contrasting all the other technologies with these two technologies, offers a framework for the problem. Additionally, the BWM's unique structure results in two vectors having only integers that avoids an underlying distance problem related with pairwise comparisons involving fractions.

### 3.3 Bayesian hierarchical model

A graphical representation of the Bayesian model can be thought of as a directed graph comprising of nodes and arcs associated with a set of probability tables. The nodes represent random variables (RVs), which can be discrete or continuous. The arcs represent directed causal relationships between variables. In the proposed model, Bayesian inference is used in the original BWM problem, where the technologies are seen as RVs from a probabilistic context, and their weights are therefore their likelihoods of occurrence. The multinomial distribution is utilized as the likelihood of the BWM's input as all the elements are integers and the Dirichlet distribution is utilized as the prior distribution of output. Since it fulfills both non-negativity as well as sum-to-one properties, the Dirichlet distribution will perfectly describe the weight vector of technologies. The posterior distribution will be Dirichlet distribution as the prior distribution was Dirichlet, and the likelihood was multinomial.

### 3.4 Proposed methodology

In this study, considering the problem context Bayesian-BWM has been chosen to identify the crucial DTs to enhance the sustainability of SC. Unlike many other MCDM techniques, Bayesian BWM does not use an arithmetic mean or a mean based on the weight of decision makers' input; instead, it is capable of using a group's collective opinion as a probability distribution.

***Step 1:*** *Identifying a set of disruptive technologies.*

In this step, a set of technologies T={t_*1,*_ t_*2,*_ t_*3 …*_ t_*n*_*}* are determined where the total number of DTs are n..

***Step 2:*** *Determining the best and the worst disruptive technologies.*

Each evaluator will detect the best (t_B_) and the worst (t_w_) technologies from the set specified as T where the total number of evaluators is L.

***Step 3:*** *Obtaining pairwise judgment between the best technology and the other disruptive technologies.*

Each evaluator conducts the pairwise judgment between the best technology and the other DTs utilizing a number “between 1 and 9”. The greater the number, the more significant the relative dominance of the technologies is. The subsequent best technology to other technologies vector is X_*B*_ *=* (x_*B1*_, x_*B2*_, *…..*, x_*Bn*_*,*). Here, x_*Bj*_ indicates the preference of the best technology over other technologies t_*j*_ *є* T.

***Step 4:*** *Obtaining the pairwise judgment between the other disruptive technologies and the worst technology.*

Similarly, each evaluator evaluates the other DTs and the worst technology utilizing a number “between 1 and 9”. The subsequent others to worst vector is X_*W*_ *=* (x_*1W*_, x_*2W*_, *…..*, x_*nW*_*)*, where x_*j*_ indicates the preference of other technologies t_*j*_ *є* T over the worst technology.

***Step 5:*** *Determining the probability distribution of weight.*

Every individual optimal weight z^*1: L*^ and aggregated optimal weight z ^*agg*^ given $X_{B}^{1:L}$ and $X_{W}^{1:L}$ are estimated, where L is the maximum number of evaluators. Equation (1) will be used to determine the joint probability distribution.

(1) $$P\left(z^{agg},z^{1:L}\;|X_{B}^{1:L},X_{W}^{1:L}\right)$$

**Fig. 2:**
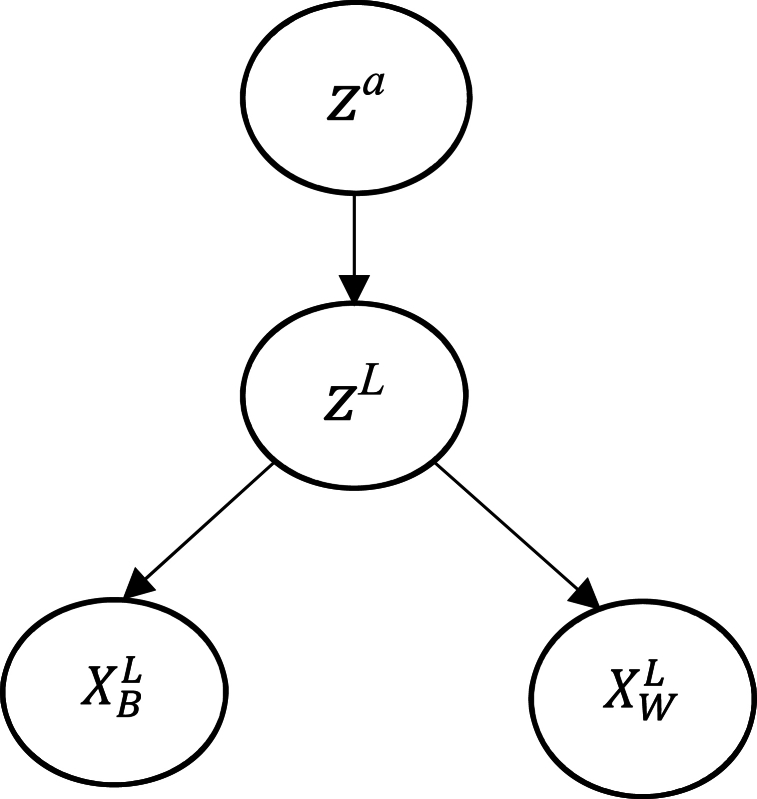
The proposed Bayesian-BWM.

To construct a Bayesian model, the first step is to determine the variables' independence and conditional dependence. The graphical models that refer to the suggested model are plotted in Fig. 2. All the nodes presented in Fig. 2 represent the variable and the directed arrow represents the conditional dependency between variables. From the figure, it can be said that z ^*L*^ depends on $X_{B}^{L}$ and $X_{W}^{L}$, whereas z ^*agg*^ depends on z ^*L*^. It is to be noted that variable z ^*L*^, $X_{B}^{L}$, and $X_{W}^{L}$ will be iterated for each evaluator.

From the above figure, the conditional independence is also clear and can be stated in the following Equation (2)**.**

(2) $$P\left(X_{B}^{L}|\;z^{agg},z^{L}\right)=P\left(X_{B}^{L}|\;z^{L}\right)$$

Applying the Bayes rule to the joint probability in Equation (1) will result in the following equation, taking into account all independence of different variables.

(3) $$P\left(z^{agg},z^{1:L}|\;X_{B}^{1:L},X_{W}^{1:L}\right)\propto P\left(X_{B}^{1:L},X_{W}^{1:L}\;|\;z^{agg},z^{1:L\backslash }\right)\;P\left(z^{agg},z^{1:L}\right)=P\left(z^{agg}\right)\;\prod_{L=1}^{L}P\left(X_{B}^{L}\;|\;z^{L}\right)\;P\;\left(X_{W}^{L}\;|\;z^{L}\right)\;P\left(z^{L}\;|\;z^{agg}\right)$$

Equation (3) has taken into consideration probability chain rule, conditional independence, and the evaluator's independent preference on each variable. The distribution of each variable in Equation (3) needs to be determined. As all the elements are integers, multinomial distribution is used to determine the probability of variables $X_{B}$ and $X_{W}$. However, $X_{B}$ is different from $X_{w}$ in the sense that where the first one shows the preference for the best technology over other technologies, the second one denotes the importance of other technologies over the worst technology. Thus, the distribution will be as:

(4) $$X_{B}^{L}|\;z^{L}∼\;multinomial\;\left(\frac{1}{\;z^{L}}\right)$$

(5) $$X_{W}^{L}\;|\;z^{L}∼\;multinomial\;\left(\;z^{L}\right)$$

A weight vector for the MCDM has to have properties such as non-negativity and sum to one in order to be valid. As a result, Dirichlet distribution is used to determine the weight. The distribution can be expressed as:

(6) $$Dir\left(z|\alpha \right)=\frac{1}{B\left(\alpha \right)}\;\prod_{j=1}^{n}\;z_{j}^{\alpha_{i=1}}$$

Here, α represents the Dirichlet distribution parameter, and z represents the optimal weight of this MCDM. For this research purpose, the Dirichlet distribution has been parameterized again considering its mean, z ^*agg*^ and concentration factor, *γ*.

(7) $$z^{L}\;|\;z^{agg}∼\;Dir\;\left(\gamma \times z^{agg}\right)$$

Equation (7) shows that the weight vector z ^*L*^ related to every evaluator should be in proximity to the mean z ^agg,^ and the closeness will be examined by the concentration parameter *γ*. The concentration factor is modeled using gamma distribution where a and b are the shape parameter.

(8) γ∼gamma(a,b)

Lastly, the prior distribution of z ^*agg*^ is derived applying an uninformative Dirichlet distribution with the factor α=1 as:

(9) $$z^{agg}\;∼\;Dir\left(1\right)$$

As (4), (5), (6), (7), (8), (9) do not create a closed loop model, a Markov Chain Monte Carlo (MCMC) sampling is performed in “Just Another Gibb Sampler (JAGS)” software to obtain the solution.

Step 6 Determining the relationship between DTs using credal ranking.

The confidence superiority as well as the relationship between a pair of technologies are determined in this step using credal ranking. Here the confidence is measured using the Dirichlet distribution. The posterior distribution of weights will aid in determining the degree of confidence in the relationships between various DTs. The confidence level will be calculated using the following definitions of credal order and ranking, which will then be utilized to construct the probabilistic hierarchical model.

**Definition 3.1**. For a pair of technology t_i_ and t_j_, a credal ordering O can be stated as shown in Equation (10)-

(10) $$O=\left(t_{i}\ldots t_{j},R,d\right)$$

Here, R represents the relationship between the technologies, and d represents the confidence between the superiority of the technologies [57].

**Definition 3.2**. For a set of technologies T={t_*1,*_t_*2,*_t_*3 …*_t_*n*_*}* the credal ranking is a series of credal orderings that comprises all pairs (t*,* t_*j*_*)* for all t_*i*_, t_*i*_ *є* T. If the sample size is M, Equation (11) can be utilized to compute the confidence that shows t_*i*_ is superior to t_*j*_ [57].

(11) $$P\left(t_{i}>t_{j}\right)=\frac{1}{M}\;\sum_{m=1}^{M}I\left(z_{i}^{{agg}_{m}}>z_{j}^{{agg}_{m}}\right)$$

## 4 Results

**Fig. 3:**
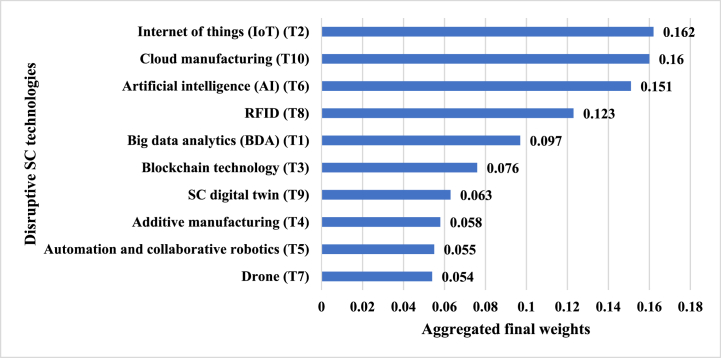
Aggregated final weights of the DTs for SCS.

This section describes the findings from the application of the proposed framework to assess DTs to achieve SSC in the context of the RMG industry of Bangladesh following the uncertain business environment. According to the very first step of the proposed Bayesian-BWM integrated framework, the DTs were considered as the alternatives for evaluation with respect to SC sustainability for the RMG industry. After collecting the responses from the evaluators, the best-to-others (BO) vectors (see Table B1 in Appendix B of supplementary file) and others-to-worst (OW) vectors (see Table B2 in Appendix B of supplementary file) were constructed. In the next step, these BO and OW vectors were modeled using the binomial distribution using Eqs. (1), (2), (3), (4), (5), and with the help of Dirichlet distribution, the aggregated final weights of the DTs were obtained using Eqs. (6), (7), (8), (9). This final weight implies the relative importance of each DT over the others to achieve sustainable SC for the RMG industry. Fig. 3 illustrates the aggregated final weights of the Decision Technologies (DTs) in the context of achieving SSC for the RMG industry in Bangladesh. These weights reflect the relative importance of each DT in contributing to SCS. From Fig. 3, it is revealed that “Internet of things (IoT) (T2)” is identified as the most important and desirable DT with the weight of 0.162 to achieve SSC for the RMG industry. The second most important DT is the “Cloud manufacturing (T10)” (weight 0.160) considering the sustainability issues of SC. The third and fourth most important and desirable DTs for achieving SSC are “Artificial intelligence (AI) (T6)” and “RFID (T8)”. “Drone (T7)” has been found to be the least important technology among the 10 DTs, with a weight of 0.054.

**Fig. 4:**
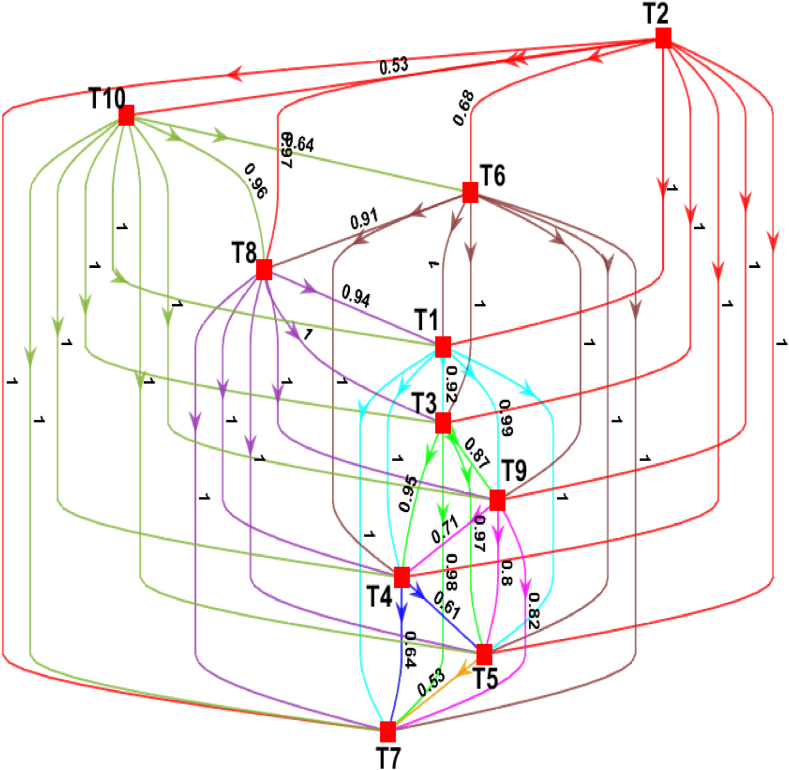
Hierarchical credal ranking of the DTs for SCS.

One of the key findings of Bayesian-BWM is the credal ranking that is a weight-directed graph to understand the interrelationship between a pair of alternatives. In this study, the confidence of selecting each DT over the others was computed using credal ranking according to Eq. (10) and Eq. (11) and visualized in Fig. 4. The credal ranking in Fig. 4 shows that “Internet of things (IoT) (T2)” is considered as the most important DT for achieving SSC, with a confidence of 0.68 against “Artificial intelligence (AI) (T6)”, with a confidence of 0.97 against “RFID (T8)” and with a confidence of 1 against “Drone (T7)”, “Automation and collaborative robotics (T5)”, “Additive manufacturing (T4)”, “SC digital twin (T9)”, “Blockchain technology (T3)” and “Big data analytics (BDA) (T1)”. On the other hand, the confidence of selecting “Internet of things (IoT) (T2)” against “Cloud manufacturing (T10)” is 0.53 indicating that these two DTs are almost equally important. From Fig. 3, it can also be seen that there is no significant difference between the weights of these two DTs. However, “Internet of things (IoT) (T2)” is still more desirable than “Cloud manufacturing (T10)” to achieve SSC for the RMG industry. The confidences of other pairs of alternatives are reasonably high except for the confidence between “Automation and collaborative robotics (T5)” and “Drone (T7)”. The confidence 0.53 between these two DTs implies that these two have almost equal importance. Overall, the credal ranking of Fig. 4 not only identifies the pivotal role of "Internet of things (IoT) (T2)" but also highlights the nuanced relationships among the DTs, shedding light on where their relative importance aligns or diverges. The visualization offers a valuable decision-making tool, enabling stakeholders in the RMG industry to make informed choices regarding technology adoption for SSC practices, taking into account both the individual DT weights and their confidence-based rankings.

## 5 Discussion

The proposed assessment framework has resulted in the relative importance of the DTs to achieve SSC for the RMG industry in an uncertain business environment. As shown in Fig. 3, “Internet of things (IoT) (T2)” is the most important and influential DT to improve the SCS for the RMG industry. Almost all the industry 4.0 and modern technologies are based on data. The use of IoT devices and sensors in the RMG industry will help to generate the data of inventory, shipping, product tracking, machine tracking, etc., that eventually help to adapt the other industry 4.0 technologies successfully and improve the overall SCS [78]. Previous studies have also found that IoT technology can improve SC by enabling real-time monitoring and tracking of products, assets, and processes that enhance visibility, transparency, operational efficiency, reduced costs, and decision-making capabilities, ultimately leading to improved operational performance [79]. Fig. 3 also shows that “Cloud manufacturing (T10)” is the second most important DT to improve SCS. The application of Cloud manufacturing will help to reduce the rate of carbon emissions in SC and improve environmental sustainability [80]. Singh et al. (2015) have found that by utilizing cloud-based platforms and services, companies can optimize resource utilization, minimize waste, and enhance energy efficiency in manufacturing processes that in turn, contributes to lower carbon emissions and a reduced environmental footprint [81]. Therefore, this technology will help the RMG industry in Bangladesh to achieve the SSC in an uncertain business environment. The hierarchical credal ranking in Fig. 4 shows that the confidence of selecting “Internet of things (IoT) (T2)” against "Cloud manufacturing (T10)" is relatively lower than the others. The “Cloud manufacturing (T10)” cannot be adopted successfully without “Internet of things (IoT) (T2)” and again, the highest benefits of “Internet of things (IoT) (T2)” cannot be obtained until it is deployed to establish cloud-based production system. “Artificial intelligence (AI) (T6)” in SC helps to improve the efficiency of the warehouse management as well as the inventory system with improved safety and relatively higher operational accuracy [82]. Abosuliman and Almagrabi, (2021) have investigated that AI techniques, such as machine learning, natural language processing, and computer vision, can bring several benefits to warehouse management such as analyzing large volumes of data, including historical inventory records, customer demand patterns, and real-time sensor data, to optimize warehouse layout, slotting, and picking processes and hence, adoption of this technology in RMG industry will also increase the SCS [83].

Over the past few years, the usage of “RFID (T8)” has been expanded surprisingly, and this technology is widely being used in inventory management, and materials handling. The use of “RFID (T8)” will reduce the unnecessary inbound and outbound transportation, and the movements of customers by providing the product availability information that eventually impacts the SCS [84]. This DT has been ranked fourth in this study. The application of “Big data analytics (BDA) (T1)” can predict the customer demands of products, energy consumption etc., and can help in formulating effective strategies accordingly [85]. Furthermore, Liu et al. [86] found that BDA can also provide insights into energy consumption patterns within the SC. Therefore, the use of “Big data analytics (BDA) (T1)” in the RMG industry will help to predict the customer demands of apparel, energy consumption, overseas orders, and the availability of resources during COVID-19. This will certainly improve the overall SCS of the RMG industry along with the customers' satisfaction and social sustainability [87]. A new digital transaction phenomenon- “Blockchain technology (T3)” is becoming popular nowadays. However, the application of this DT in SC is still very limited in the emerging economy like Bangladesh. “Blockchain technology (T3)” will help the RMG industry to digitize their SC system with the highest level of tracking facilities from production to delivery [88] and ensures the highest level of security in information transfer, transaction, verification, etc., without the help of third parties. In addition, this technology is also economically sustainable as it helps to reduce SC costs of the organizations and improves business performance. One of the latest DTs in SC, “SC digital twin (T9)” simulates the actual SC and provides real-time data [45]. Thus, the adoption of this technology will help the RMG industry to reduce the uncertainties and risks across the SC as well as minimize the human interventions in various SC activities. However, successful implementation of this technology requires huge knowledge and training of people as well as substantial costs [89], and therefore, this technology is less sustainable than the other DTs from the social and economic point of views. From Fig. 3, it can also be seen that “SC digital twin (T9)” is ranked seventh among the 10 SC technologies from the sustainability point of view.

“Additive manufacturing (T4)” and “Automation and collaborative robotics (T5)” have shifted the total SC to an extraordinary level with higher flexibility and agility. Previous studies have highlighted the potential of automation and additive manufacturing in enabling on-demand manufacturing, reducing inventory levels, and shortening SCs [90]. Although these technologies are very influential in increasing the SC performances, the rates of carbon footprint of these technologies are higher than the traditional technologies [91]. Moreover, successful implementation of these technologies requires enough investment and adequate training. Therefore, in this study, “Additive manufacturing (T4)” and “Automation and collaborative robotics (T5)” are found to be less desirable and significant than the other DTs to achieve SSC for the RMG industry. The use of “Drone (T9)” in SC activities is becoming very popular nowadays. However, there are some issues with the use of drones in the RMG industry of Bangladesh. There exist some government regulations in Bangladesh regarding the use of drones because of privacy issues. The application of drones in the SC activities also requires substantial investment, and hence, this technology is less sustainable in an emerging economy like Bangladesh.

This study has considered the context of emerging economies to evaluate the DTs to achieve SSC for the RMG industry. The proposed framework using Bayesian-BWM has been used to obtain a more logical ranking of these technologies in a probabilistic sense. This framework also resulted in a hierarchical credal ranking model with a confidence level that has revealed the interrelation among these technologies explicitly. The theoretical and practical implications of the proposed framework have been discussed in the following subsections.

### 5.1 Validation of results

The disruptive SC technology and hierarchical credal ranking were subjected to validation through focus group discussions involving 12 industrial managers who served as industry practitioners. The selection process considered their years of experience, current organizational affiliation, and expertise area, with a focus on SC technologies and sustainability. The profile of experts for validation has been provided in Table C1 of Appendix C. The discussions occurred in two phases. In the first phase, the participants were given the ranking of DTs for SCS and were asked to validate it. After the discussion, a consensus was reached among the participants regarding the ranking of DTs. In the second phase, the participants were presented with the credal ranking, which included the confidence of experts in selecting one DT over another. They were asked for their opinions on the appropriateness of the confidence level of experts and its relevance to the RMG industry context. The participants expressed that they considered the ranking of DTs with expert confidence levels to be appropriate and relevant in the current context. Overall, this phase of the study helped validate and refine the identified DTs for SCS and provided a deeper understanding of the relationships among them.

### 5.2 Theoretical implication

The application of DTs in the RMG industry is not yet widespread in developing countries, even in developed countries. In Bangladesh's garments industry, a new realm of possibility can be created via revolutionary transformation brought about by disseminating DTs. Moreover, in the fourth industrial revolution, the garment industry must focus on three-pronged strategies: social, economic, and environmental. It is not possible to achieve sustainability without transforming the massive data acquired via automation and digitalization into essential information [92]. Furthermore, efficiency, sustainability, and quick responsiveness to client requirements are among the difficulties that RMG face in today's competitive and uncertain business environment. Adopting DT allows the RMG industry to adapt to changing market demands and embrace innovation. For example, e-commerce platforms and digital marketing strategies enable companies to reach a wider customer base and respond to trends quickly. The adoption of DT in the readymade garments industry requires a skilled workforce. Companies need to invest in upskilling and reskilling their employees to effectively operate and maintain advanced technologies. This transformation can lead to the creation of new job roles and opportunities, promoting a more tech-savvy and digitally skilled workforce.

Previous literature has explicitly addressed the inherent capabilities of DTs in different industries. For instance, the existing studies have emphasized the recent development of IoT which has resulted in the connectivity of various technologies, allowing for better monitoring and coordination among business partners [13]. In RMG, IoT might help with many facets of fashion design, development, and production. Moreover, using cloud manufacturing to carry out operations improves the management experience [93]. Likewise, large quantities of work need a high degree of efficiency in manufacturing equipment to ensure consistency and precision. AI may be used to solve this and provide numerous advantages such as labor savings, cycle reduction, better components quality, enhanced safety, productivity, and efficiency [94]. In addition, the usage of RFID technology aids in the tracking of goods throughout the manufacturing process and can aid in the automation of the garment manufacturing process [44].

From a theoretical viewpoint, this research facilitates the development of a systematic judgment approach for DTs in SSC. The integrated Bayesian-BWM approach has been used to achieve the stated goals while also embedding sustainability issues. This proposed approach differs from other traditional MCDM methods in that it ranks not only the critical DTs but also demonstrates the probabilistic hierarchical relationships between all the pairs of DTs that show the degree to which one DT is superior to other DTs. Moreover, this research contributes to the current literature by integrating probabilistic inference in MCDM to compute the aggregated weight using a probability distribution. Ergo, this research would aid in the acceleration of technology adoption for sustainable production in developing countries.

### 5.3 Practical implication

In terms of enhancing the sustainability of SC, the suggested Bayesian-BWM approach offers a rational, systematic, and convenient technique to assist policymakers and industry professionals of RMG in achieving sustainable adoption of DTs. The DTs evaluation approach can be extended to SC technology management in a practical way. Managers and policymakers can measure DTs on the three sustainability dimensions using the proposed Bayesian-BWM approach to decide sustainability targets and which DTs to adopt or encourage for sustainable production via policy interventions [94]. By focusing on important DTs, the recently introduced Bayesian-BWM system is expected to direct SC and operations managers to improve efficiency and ensure sustainable production. Therefore, this research would aid managers and business experts in taking constructive measures, economically viable and ecologically sound decisions with supporting information for DTs adoption in the SC.

The proposed integrated Bayesian-BWM model to prioritize the critical DTs will aid policymakers to adopt technology in their SC and achieve SDGs. The study reveals that “Internet of things (IoT) (T2)”, “Cloud manufacturing (T10)”, are the most significant technologies which have a high potential to improve the SSCM operations models and enhance the efficiency of organizations in emerging economies. Industry, innovation, and infrastructure (SDG 9) can be achieved by adopting IoT in organizations of emerging economies. As “cloud manufacturing (T10)” technology will connect all the stakeholders through a collaborative and strategic approach towards various SC activities, it will aid to achieve SDG 9 and partnerships for the goals (SDG 17). Furthermore, implementing cloud manufacturing technologies would aid climate action (SDG 13) by reducing carbon emissions and energy consumption. “RFID (T8)” technology adoption in organizations will ensure using working capital effectively, reducing operational and labor costs while mitigating the security risk. This technology adoption will lead to achieving responsible consumption and production (SDG 12). “Artificial intelligence (T6)”, “Big data analytics (T1)”, “Blockchain technology (T3)”, “SC digital twin (T9)” will help to achieve sustainable cities and communities (SDG 11) and SDG 9. Moreover, “Additive manufacturing (T4)” and “Automation and collaborative robotics (T5)” will speed up the manufacturing system with improved process systems in traditional production systems hence achieving SDG 9. Overall, by having an awareness of the various crucial DTs, along with their impact on SDGs, managers can develop more efficient strategies to increase their overall operational excellence in sustainable production.

## 6 Conclusion

The assessment of DT is critical for the emerging economies for sustainability journey. Identifying and evaluating the most important DTs is crucial for implementing long-term plans and sustaining an uncertain business environment. Therefore, the Bayesian-BWM model for disruptive SC technologies assessment is presented in this paper. This research has presented a Bayesian-BWM and confirmed its applicability by collecting input from the experts of Bangladesh. The key objective of this study was to assess the DTs to increase the sustainability of the SC from a sustainable perspective for an emerging economy like Bangladesh. Based on the extant literature, 10 DTs were initially identified. After applying Bayesian-BWM, "Internet of things (IoT) (T2)" is found as the most influential DT, followed by “Cloud manufacturing (T10)” and “Artificial intelligence (AI) (T6)”. To evaluate the viability of disruptive SC innovations, this study took into account the emerging economy context. Most importantly, the Bayesian-BWM was used to rank these SC technologies in a more logical order. In order for one to be interested in the group's collective opinion, the Bayesian-BWM is an emerging tool in group decision-making, where one could also check the weight rating in a probable way. It is, nevertheless, critical to note the limitations to this study. The findings of this study may be limited to the investigated phenomenon and its context (e.g., geographical, historical etc.). The study has solely concentrated the efforts on evaluating DTs in the RMG industry. In the future, other industries may be considered to evaluate DT in their SC to enhance sustainability. Moreover, the location of outlines i.e., the positioning of the best and worst alternatives within a set of options and consistency measures of different BWM need to be explored in future.

This research presents a number of different directions for future research. The suggested approach can be integrated with other MCDM approaches like fuzzy AHP, rough AHP, DEMATEL, neutrosophic DEMATEL, etc. More expert input can be taken as well as experts can be chosen from different regions. The method is expected to be implemented for other country contexts as well. Every SC functioning in any industrial or country context should adopt DTs to take a competitive advantage and improve the resilience capacity. For example, managers of an Indian-based organization can examine DTs and use the model this paper has suggested to prioritize and evaluate their available DTs. Therefore, managers and policymakers from any large, small, and medium enterprises in any emerging country can follow the generic steps used in this paper.

## Data availability statement

The data used in this study are included in the paper and supplementary materials.

## CRediT authorship contribution statement

**Humaira Nafisa Ahmed:** Writing – review & editing, Writing – original draft, Software, Methodology, Investigation, Formal analysis, Data curation, Conceptualization. **Sayem Ahmed:** Writing – review & editing, Writing – original draft, Software, Methodology, Investigation, Formal analysis, Data curation, Conceptualization. **Tazim Ahmed:** Writing – review & editing, Writing – original draft, Software, Methodology, Investigation, Formal analysis, Data curation, Conceptualization. **Hasin Md Muhtasim Taqi:** Writing – review & editing, Writing – original draft, Software, Methodology, Investigation, Formal analysis, Data curation, Conceptualization. **Syed Mithun Ali:** Writing – review & editing, Writing – original draft, Visualization, Supervision, Resources, Methodology, Investigation, Conceptualization.

## Declaration of competing interest

The authors declare that they have no known competing financial interests or personal relationships that could have appeared to influence the work reported in this paper.
