# Supplementary material for: Disruptive supply chain technology assessment for sustainability journey: A framework of probabilistic group decision making
Source: Heliyon. 2024 Feb 8;10(4):e25630. doi: 10.1016/j.heliyon.2024.e25630 (PMC10878870; doi:10.1016/j.heliyon.2024.e25630)
Supplement: Multimedia component 1 [file mmc1.docx]

**Appendix A**

Questionnaire

1. Information of the respondent:

1. Name:
2. Name of organization:
3. Organization type:
4. Designation:
5. Experience:
6. Expertise:

2. Please, select the most important and the least important supply chain (SC) technologies in the context of Bangladesh from the sustainability point of view.

**Table A1**: Identification of the best and worst technology

| **Factors Code** | **Factors Name** | **Most important (Please check)** | **Least important**  **(Please check)** |
| --- | --- | --- | --- |
| T1 | Big data analytics (BDA) |  |  |
| T2 | Internet of things (IoT) |  |  |
| T3 | Blockchain technology |  |  |
| T4 | Additive manufacturing |  |  |
| T5 | Automation and collaborative robotics |  |  |
| T6 | Artificial intelligence (AI) |  |  |
| T7 | Drone |  |  |
| T8 | RFID |  |  |
| T9 | SC digital twin |  |  |
| T10 | Cloud manufacturing |  |  |

3. Please, score the precedence of the best technology over all other technologies using the scale between 1 to 9. Here, 1 refers to equal precedence and 9 refers to very high precedence.

**Table A2**: Precedence score of the best technology over other technologies

| Best  technology | T1 | T2 | T3 | T4 | T5 | T6 | T7 | T8 | T9 | T10 |
| --- | --- | --- | --- | --- | --- | --- | --- | --- | --- | --- |
|  |  |  |  |  |  |  |  |  |  |  |

4. Please, score the precedence of all technologies over the worst technology using the scale between 1 to 9. Here, 1 refers to equal precedence and 9 refers to very high precedence.

**Table A3**: Precedence scores of other technologies over the worst technology

| Worst technology |  |
| --- | --- |
| T1 |  |
| T2 |  |
| T3 |  |
| T4 |  |
| T5 |  |
| T6 |  |
| T7 |  |
| T8 |  |
| T9 |  |
| T10 |  |

**Appendix B**

**Table B1:** Best-to-Others (BO) vectors for the 10 evaluators

| Expert | Best  technology | T1 | T2 | T3 | T4 | T5 | T6 | T7 | T8 | T9 | T10 |
| --- | --- | --- | --- | --- | --- | --- | --- | --- | --- | --- | --- |
| E1 | T2 | 7 | 1 | 5 | 6 | 8 | 5 | 9 | 4 | 6 | 3 |
| E2 | T10 | 4 | 2 | 6 | 7 | 8 | 2 | 9 | 3 | 5 | 1 |
| E3 | T2 | 6 | 1 | 7 | 9 | 5 | 3 | 4 | 5 | 8 | 2 |
| E4 | T2 | 3 | 1 | 6 | 4 | 8 | 4 | 9 | 2 | 7 | 5 |
| E5 | T6 | 4 | 5 | 5 | 9 | 7 | 1 | 6 | 3 | 6 | 2 |
| E6 | T2 | 5 | 1 | 6 | 7 | 9 | 2 | 5 | 4 | 8 | 3 |
| E7 | T10 | 6 | 4 | 7 | 8 | 6 | 3 | 9 | 5 | 8 | 1 |
| E8 | T2 | 5 | 1 | 6 | 8 | 8 | 3 | 9 | 4 | 7 | 2 |
| E9 | T2 | 4 | 1 | 5 | 7 | 9 | 2 | 8 | 3 | 6 | 2 |
| E10 | T6 | 5 | 6 | 6 | 8 | 7 | 1 | 9 | 3 | 7 | 2 |

**Table B2:** Others-to-Worst (OW) vectors for the 10 evaluators

| Expert | E1 | E2 | E3 | E4 | E5 | E6 | E7 | E8 | E9 | E10 |
| --- | --- | --- | --- | --- | --- | --- | --- | --- | --- | --- |
| Worst technology | T7 | T7 | T4 | T7 | T4 | T5 | T7 | T7 | T5 | T7 |
| T1 | 3 | 6 | 5 | 7 | 6 | 5 | 4 | 5 | 6 | 5 |
| T2 | 9 | 8 | 9 | 9 | 5 | 9 | 6 | 9 | 9 | 4 |
| T3 | 5 | 3 | 3 | 5 | 4 | 4 | 3 | 4 | 5 | 4 |
| T4 | 4 | 3 | 1 | 6 | 1 | 3 | 2 | 2 | 3 | 2 |
| T5 | 2 | 2 | 5 | 2 | 3 | 1 | 4 | 3 | 1 | 3 |
| T6 | 4 | 7 | 7 | 6 | 9 | 8 | 7 | 7 | 8 | 9 |
| T7 | 1 | 1 | 6 | 1 | 4 | 6 | 1 | 1 | 3 | 1 |
| T8 | 6 | 7 | 5 | 7 | 7 | 5 | 5 | 6 | 7 | 7 |
| T9 | 3 | 5 | 2 | 3 | 4 | 2 | 2 | 3 | 4 | 3 |
| T10 | 7 | 9 | 7 | 5 | 8 | 7 | 9 | 8 | 7 | 8 |

**Appendix C**

**Table C1:** Profile of experts for validation of the findings

| **Expert** | **Designation** | **Experience** | **Expertise** |
| --- | --- | --- | --- |
| Expert 1 | Head of Supply Chain | 25 years | Supply chain management and sustainability |
| Expert 2 | General Manager, Logistics | 18 years | Logistics and technology |
| Expert 3 | Chief Supply Chain Officer | 17 years | Supply chain and operations management |
| Expert 4 | General Manager, Supply Chain | 18 years | Supply chain sustainability |
| Expert 5 | General Manager, Supply Chain | 20 years | Supply chain sustainability |
| Expert 6 | Head of Supply Chain and Operation | 16 years | Supply chain management |
| Expert 7 | Deputy General Manager, Supply Chain | 16 years | Supply chain technology and industry 4.0 |
| Expert 8 | General Manager, Procurement and Planning | 18 years | Supply chain and industry 4.0 |
| Expert 9 | Senior Logistics Manager | 15 years | Logistics and operations management |
| Expert 10 | Deputy Manager, IT | 11 years | Supply chain management and industry 4.0 |
| Expert 11 | Senior Manager, ERP & IT | 10 years | Supply chain management and industry 4.0 |
| Expert 12 | General Manager, Supply Chain | 11 years | Supply chain management and industry 4.0 |
